# T-cell count and T-cell telomere length in patients with severe COVID-19

**DOI:** 10.3389/fimmu.2024.1356638

**Published:** 2024-03-14

**Authors:** Bryan D. Kraft, Simon Verhulst, Tsung-Po Lai, Bruce A. Sullenger, Yunfei Wang, Wes Rountree, Lingye Chen, Christopher W. Woods, Thomas N. Denny, Abraham Aviv

**Affiliations:** ^1^ Division of Pulmonary, Allergy, and Critical Care Medicine, Duke University Medical Center, Durham, NC, United States; ^2^ Groningen Institute for Evolutionary Life Sciences, University of Groningen, Groningen, Netherlands; ^3^ Center of Human Development and Aging, New Jersey Medical School, Rutgers, The State University of New Jersey, Newark, NJ, United States; ^4^ Department of Surgery, Duke University Medical Center, Durham, NC, United States; ^5^ Duke Human Vaccine Institute, Durham, NC, United States; ^6^ Center for Infectious Disease Diagnostics and Innovation, Duke University Medical Center, Durham, NC, United States; ^7^ Department of Medicine, Durham Veterans Affairs Health Care System, Durham, NC, United States; ^8^ Department of Pediatrics, New Jersey Medical School, Rutgers, The State University of New Jersey, Newark, NJ, United States

**Keywords:** SARS-CoV-2, COVID-19, T-lymphocytes, lymphopenia, sex, aging, telomere

## Abstract

Lymphocyte telomere length (TL) is highly variable and shortens with age. Short telomeres may impede TL-dependent T-cell clonal expansion with viral infection. As SARS-CoV-2 infection can induce prolonged and severe T-cell lymphopenia, infected adults, and particularly older adults with short telomeres, may display severe T-cell lymphopenia. To examine the relationship between T-cell TL parameters and T-cell counts, we studied 40 patients hospitalized with severe COVID-19. T-cells were isolated from lymphocytes, counted using flow cytometry, and their TL parameters were measured using the Telomere Shortest Length Assay. The cohort (median age = 62 years, 27% female) was racially and ethnically diverse (33% White, 35% Black, and 33% Other). On intensive care unit study day 1, T-cell count (mean=1.03 x10^9^/L) was inversely related to age (p=0.007) and higher in females than males (p=0.025). Mean TL was 3.88 kilobases (kb), and 45.3% of telomeres were shorter than 3 kb. Using multiple regression analysis and adjusting for age and sex, T-cell count decreased with increased proportion of T-cell telomeres shorter than 3 kb (p=0.033) and increased with mean TL (p=0.052). Our findings suggest an association between the buildup of short telomeres within T-cells and explain in part reduced peripheral blood T-cell counts in patients with severe COVID-19. Shortened T-cell telomeres may be a risk factor for COVID-19-associated T-cell lymphopenia.

## Introduction

Transient lymphopenia is common in acute viral respiratory infections. However, SARS-CoV-2 infection may induce prolonged and severe lymphopenia characterized by a marked decrease in the T-cell count, which is associated with COVID-19 severity (1). Offsetting COVID-19 T-cell lymphopenia, or recovering from it, likely requires rapid and massive T-cell clonal expansion (2), which is telomere length (TL)-dependent (3). Leukocyte TL, which reflects the overall TL of different leukocyte lineages, is highly variable across individuals from birth and shortens with age (4). Consequently, some adults, but particularly older adults, may have T-cells with limited ability for clonal expansion due to shortened telomeres, making these individuals more vulnerable to COVID-19-related T-cell lymphopenia and severe COVID-19 (2, 5). This proposition is supported by modeling demonstrating that a decline in TL-dependent T-cell clonal expansion capacity with age may contribute to increased COVID-19 mortality in older persons (2). It is also supported by the following findings: First, adults with short leukocyte telomeres, measured before contracting COVID-19, were more likely to experience severe COVID-19, irrespective of age (6). Second, lymphocyte count in hospitalized elderly COVID-19 patients correlated with leukocyte TL parameters (7).

These theoretical and empirical findings suggest that TL-dependent T-cell proliferation contributes to COVID-19 pathogenesis. However, previous studies have examined TL in leukocytes (principally neutrophils and lymphocytes) and peripheral blood mononuclear cells (PBMCs, principally lymphocytes). No study has probed the relationship between T-cell TL parameters and T-cell counts in COVID-19. To address this gap, we studied the relationship between TL parameters and T-cell count in hospitalized patients with severe COVID-19.

## Methods

### Enrollment

The Duke University COVID-19 Intensive Care Unit (ICU) Biorepository is an observational cohort study of critically ill patients with severe COVID-19 admitted to the ICU. The study was approved by the Duke University institutional review board (#Pro00101196). Enrolled patients met the following criteria: (i) positive SARS-CoV-2 test of nasopharyngeal swab by reverse transcriptase polymerase chain reaction (RT-PCR), (ii) age 18 years or older, and (iii) admission to the ICU.

### Data collection

Clinical data were obtained from the electronic medical record. PBMCs were isolated from whole blood (citrated CPT tubes, Becton Dickinson) on the day of enrollment in the study in the ICU. T-cells (CD3+) were isolated from PBMCS using immunomagnetic cell separation (MACSxpress Technology, Miltenyi) and T-cell DNA was extracted using Qiagen kits. Flow cytometry of whole blood was performed (B53309, Beckman-Coulter).

### Telomere measurements

Each human somatic cell nucleus contains 92 telomeres at the ends of the 23 chromosome pairs. The signal triggering the cessation of somatic cell replication originates from the shortest telomeres rather than the mean TL (8), but the shortest telomeres cannot be detected using conventional techniques. We therefore used the Telomere Shortest Length Assay (TeSLA) to enumerate and measure the lengths of telomeres, including those shorter than 3 kilobase (kb) (9).

### Statistical analysis

Data are presented as median (quartile 1, quartile 3) for time intervals, or mean (standard deviation) for other variables. TL data were log_10_ transformed to normalize the distribution. Multiple regression analysis was performed using maximum likelihood estimation in SAS. We assumed a Gaussian error distribution, which was confirmed through visual inspection of the residuals. We fitted two models in which TL was characterized by either the proportion of telomeres shorter than 3 kb (expressed as a percent of all telomeres) or by mean TL (expressed in kb). In both models we included age (covariate) and sex (factor) as fixed effects because both variables are known to affect TL (4).

## Results

Forty patients with severe COVID-19 were enrolled in 2020-2021 comprising 29 males and 11 females, median age 62 years, and 33% White, 35% Black, and 33% Other (**Table 1**). COVID-19 diagnosis was confirmed by RT-PCR at a median of 11 days prior to ICU admission, and subjects were enrolled at a median of 5 days after admission to the ICU.

**Table 1:** Baseline Characteristics.

Baseline Characteristics	Subject Cohort (n=40)
Age (years)	62 (56, 72)
Sex	
Male	29 (73%)
Female	11 (27%)
Race	
White	13 (33%)
Black or African American	14 (35%)
Other	13 (33%)
Ethnicity	
Non-Hispanic/Latino	28 (70%)
Hispanic/Latino	11 (28%)
Unknown	1 (3%)
Comorbidities	
Diabetes mellitus	20 (50%)
Malignancy	4 (10%)
Chronic immunosuppression	2 (5%)
Autoimmune disease	3 (8%)
Chronic lung disease	10 (25%)
Chronic kidney disease	6 (15%)
Chronic heart disease	8 (20%)
Body mass index	32 (27, 38)
First symptoms to enrollment (days)^a^	16 (11, 22)
First RT-PCR to enrollment (days)	11 (8, 18)
Hospitalization to enrollment (days)	5 (3, 7)
T-cell count (x10^9^/L)	1.03 (0.69)
TeSmTL (kb)	3.88 (0.48)
TeS3kb (%)	45.3 (7.2)

kb, kilobase pairs; RT-PCR, reverse transcriptase polymerase chain reaction; TeSmTL, mean telomere length; TeS3kb, telomeres shorter than 3 kb. Data presented as median (Q1, Q3), mean (standard deviation), or count (%). ^a^Data missing in 13 of the 40 subjects.

**Figure 1:**
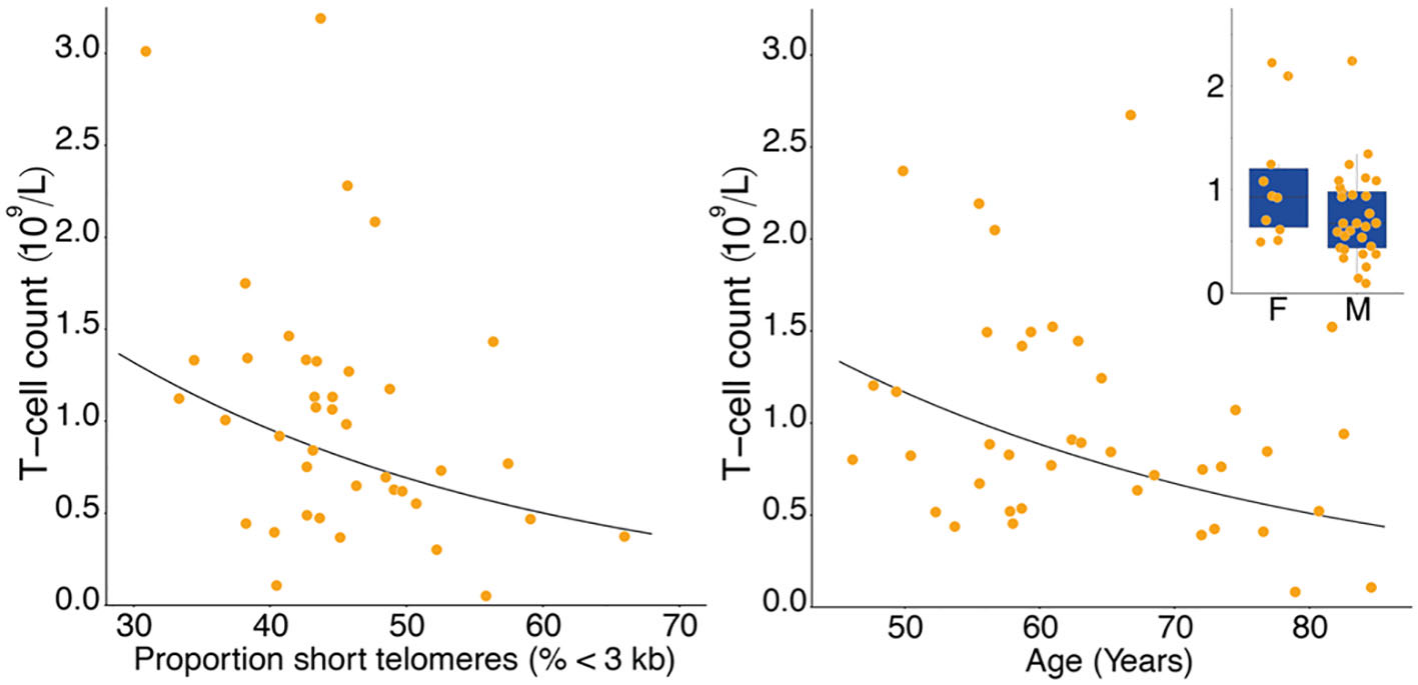
Lower T-cell count with higher proportion of T-cell short telomeres (left panel), and lower T-cell count with age (right panel). Inset shows lower T-cell count in males (M) than in females (F). Data in the right panel were adjusted for proportion of short telomeres and sex, data in the inset were adjusted for proportion of short telomeres and age.

T-cell and TL parameters were measured in samples collected on the day of enrollment: Mean (SD) T-cell count (x10^9^/L) was 1.03 (0.69), TL was 3.88 (0.48) kb, and the proportion of telomeres shorter than 3 kb was 45.3% (7.2%) (**Table 1**). T-cell count was inversely related to age (p=0.007) and higher in females than males (p=0.025). (**Table 2**, **Figure 1**). After adjusting for age and sex, multiple regression analysis showed that T-cell count decreased with increased proportion of T-cell telomeres shorter than 3 kb (p=0.033) (**Table 2**, **Figure 1**) and increased with mean TL (p=0.052) (**Table 2**, **Figure 1**). Interactions of age and sex with the T-cell TL parameters did not reach statistical significance, indicating effects of age, sex, and T-cell TL parameters on T-cell count were independent and additive.

**Table 2:** T-cell count (log transformed) in relation to (A) proportion of telomeres shorter than 3 kilobase (R^2^ = 0.34), and (B) mean telomere length (R^2^ = 0.33), age, and sex by maximum likelihood estimation.

A.	Estimate (SE)	T	p
Intercept	10.25 (0.38)	26.85	<0.0001
Proportion <3 kb (%)	-0.014 (0.0063)	-2.22	0.033
Age (years)	-0.012 (0.0042)	-2.85	0.007
Sex	0.23 (0.10)	2.34	0.025

B.	Estimate (SE)	T	p
Intercept	8.93 (0.45)	19.72	<0.0001
Mean length (kb)^1^	0.19 (0.095)	2.01	0.052
Age (years)	-0.013 (0.10)	-3.06	0.004
Sex	0.25 (0.10)	2.45	0.019

Sex estimate represents females relative to males. ^1^Mean length includes telomeres shorter than 3 kilobases. Abbreviations: kb, kilobase; SE, standard error.

## Discussion

Our findings reveal associations of T-cell count with TL parameters of T-cell telomeres in patients with severe COVID-19. These associations reflect a dynamic outcome where TL-dependent T-cell clonal expansion is occurring during the progression of SARS-CoV-2 infection. T-cell clonal expansion during the infection likely serves two essential purposes: It is a cardinal feature of the adaptive immune response, generating effector/memory T cells against the virus, and it helps to recover from or prevent T-cell lymphopenia due to the infection.

Our findings support the hypothesis that short telomeres within T-cells may hinder their ability to undergo adequate clonal expansion in response to SARS-CoV-2 infection (2, 5). Short telomeres may weaken both the ability of clearing SARS-CoV-2 and to restore T-cell counts to normal levels: The outcome is severe COVID-19-associated T-cell lymphopenia. We attribute the ability of showing the TL-T-cell count connection in COVID-19 patients to the unique capabilities of TeSLA for detecting and quantifying short telomeres (5, 7).

Leukocyte TL parameters are associated with age, i.e., mean TL shortens with age, while the proportion of telomeres shorter than 3 kb increases with age (4). Moreover, adjusted for age, mean leukocyte TL is longer while the percentage of telomeres shorter than 3 kb is lower in females than males (4). Yet we observed the associations between T-cell counts and TL parameters regardless of age and sex.

This is the first publication reporting the use of TeSLA for measuring T-cell TL parameters. In our study, the median age of participants was 62 years, with mean TL and proportion of telomeres shorter than kb standing at 3.88 kb and 45.3%, respectively. These estimates are close to observations in a recent study examining TL parameters in leukocytes of healthy individuals from the general population, mean leukocyte TL at age 62 years was 3.8 kb, with the proportion of telomeres shorter than 3 kb recorded at 43% (4). Given that TL differences across leukocyte lineages within individuals are considerably smaller than differences among individuals (10), robust correlations exist between TL parameters in leukocytes and those in leukocyte lineages. Consequently, our findings suggest that leukocyte TL parameters could serve as approximations for TL parameters in leukocyte lineages in large-scale, population-based studies. It’s worth noting in this regard that the phenomenon of ‘synchrony’ in TL across leukocyte lineages (10) may not apply in some disease states. However, this doesn’t seem to be the case for COVID-19, based on T-cell TL measurements. Nevertheless, it would be beneficial to explore the relationship between TL parameters, as measured by TeSLA, in the two types of T cells, CD4 and CD8 T cells.

We acknowledge the study limitations. First, the investigation is a small single center cohort study, and the results should be confirmed in larger multicenter studies. Second, we did not measure T-cell TL parameters in non-critically ill patients with COVID-19 and therefore cannot comment how T-cell TL may relate to T-cell counts in less severe illness.

In conclusion, patients with severe COVID-19 display associations of T-cell TL parameters with T-cell counts that are independent of sex and age. These findings further support the tenet that T-cell TL parameters play a role in the pathogenesis of COVID-19.

## Data availability statement

The raw data supporting the conclusions of this article will be made available by the authors, without undue reservation.

## Ethics statement

The studies involving humans were approved by Duke University Institutional Review Board. The studies were conducted in accordance with the local legislation and institutional requirements. Written informed consent for participation in this study was provided by the participants’ legal guardians/next of kin.

## Author contributions

BK: Conceptualization, Investigation, Funding acquisition, Project administration, Resources, Writing – original draft, Writing – review & editing. SV: Formal Analysis, Writing – review & editing. BS: Conceptualization, Investigation, Resources, Writing – review & editing. YW: Formal Analysis, Writing – review & editing. WR: Formal Analysis, Writing – review & editing. CW: Conceptualization, Investigation, Funding acquisition, Writing – review & editing. TD: Conceptualization, Investigation, Funding acquisition, Project administration, Resources, Writing – review & editing. AA: Conceptualization, Investigation, Funding acquisition, Project administration, Resources, Writing – original draft, Writing – review & editing. T-PL: Investigation, Methodology, Resources, Writing - review & editing. LC: Investigation, Project administration, Resources, Writing - review & editing.

## References

[1] Chen Z John Wherry E. T cell responses in patients with COVID-19. Nat Rev Immunol. (2020) 20:529–36. doi: 10.1038/s41577-020-0402-6 PMC7389156 32728222

[2] Anderson JJ Susser E Arbeev KG Yashin AI Levy D Verhulst S. Telomere-length dependent T-cell clonal expansion: A model linking ageing to COVID-19 T-cell lymphopenia and mortality. EBioMedicine. (2022) 78:103978. doi: 10.1016/j.ebiom.2022.103978 35367774 PMC8970968

[3] Patrick MS Cheng NL Kim J An J Dong F Yang Q. Human T cell differentiation negatively regulates telomerase expression resulting in reduced activation-induced proliferation and survival. Front Immunol. (2019) 10:1993. doi: 10.3389/fimmu.2019.01993 31497023 PMC6712505

[4] Lai TP Verhulst S Savage SA Gadalla SM Benetos A Toupance S. Buildup from birth onward of short telomeres in human hematopoietic cells. Aging Cell. (2023) 22:e13844. doi: 10.1111/acel.13844 37118904 PMC10265151

[5] Aviv A. Telomeres and COVID-19. FASEB J. (2020) 34:7247–52. doi: 10.1096/fj.202001025 PMC7276714 32427393

[6] Wang Q Codd V Raisi-Estabragh Z Musicha C Bountziouka V Kaptoge S. Shorter leukocyte telomere length is associated with adverse COVID-19 outcomes: A cohort study in UK Biobank. EBioMedicine. (2021) 70:103485. doi: 10.1016/j.ebiom.2021.103485 34304048 PMC8299112

[7] Benetos A Lai TP Toupance S Labat C Verhulst S Gautier S. The nexus between telomere length and lymphocyte count in seniors hospitalized with COVID-19. J Gerontol A Biol Sci Med Sci. (2021) 76:e97–e101. doi: 10.1093/gerona/glab026 33528568 PMC7929343

[8] Hemann MT Strong MA Hao LY Greider CW. The shortest telomere, not average telomere length, is critical for cell viability and chromosome stability. Cell. (2001) 107:67–77. doi: 10.1016/S0092-8674(01)00504-9 11595186

[9] Lai TP Zhang N Noh J Mender I Tedone E Huang E. A method for measuring the distribution of the shortest telomeres in cells and tissues. Nat Commun. (2017) 8:1356. doi: 10.1038/s41467-017-01291-z 29116081 PMC5676791

[10] Kimura M Gazitt Y Cao X Zhao X Lansdorp PM Aviv A. Synchrony of telomere length among hematopoietic cells. Exp Hematol. (2010) 38:854–9. doi: 10.1016/j.exphem.2010.06.010 PMC3142672 20600576

